# Ebola Stigma and Its Impact on Outbreak Control: Lessons From Key Informant Interviews in Central Uganda

**DOI:** 10.1111/tmi.70014

**Published:** 2025-08-03

**Authors:** Amy Paterson, Olive Kabajaasi, Francess Adlard, Kkunsa Hadson Dimitrios, Ashleigh Cheyne, Yasin Ssewankambo, David Kaggwa, Piero Olliaro, Nathan Kenya‐Mugisha, Amanda Rojek

**Affiliations:** ^1^ Pandemic Sciences Institute University of Oxford Oxford UK; ^2^ Walimu Kampala Uganda; ^3^ School of Medicine and Biomedical Sciences University of Oxford UK; ^4^ St Nikolas Pharmacy Kampala Uganda; ^5^ Entebbe Isolation Centre Entebbe Uganda; ^6^ Fort Portal Regional Referral Hospital Fort Portal Uganda

**Keywords:** Ebola disease, framework analysis, outbreak response, public health preparedness, qualitative research, social determinants of health, stigma, Sudan ebolavirus

## Abstract

**Objectives:**

The 2022 outbreak of Sudan ebolavirus in central Uganda was the country's largest in two decades. It was accompanied by reports of stigma towards affected individuals, households and communities. The objectives of this study were to (1) describe how Ebola disease stigma emerged and manifested during the 2022 Sudan ebolavirus outbreak in central Uganda, (2) examine its impacts, including on outbreak control and (3) identify insights that could inform stigma reduction strategies in future outbreaks.

**Methods:**

We conducted qualitative in‐depth interviews with 12 key informants involved in the Ebola disease outbreak response using Microsoft Teams. Participants included frontline healthcare workers, burial team members, psychosocial support staff, survivor programme staff, village health team members, local outbreak response leadership and Ebola survivors. Transcribed interviews were coded in NVivo Release 1.7.2 and analysed using framework analysis.

**Results:**

Contextual drivers of stigma included mistrust of authorities, limited knowledge about the disease and conspicuous survivor follow‐up. These drivers fuelled negative thoughts and emotions, predominantly blame and fear. Interviewees described how stigma manifested as negative attitudes, verbal and physical harm, unwarranted avoidance and structural disadvantage, which persisted beyond the outbreak itself. Stigma was seen to impact outbreak control by discouraging symptom reporting, delaying care‐seeking and exacerbating workforce shortages in clinical centres. Factors that mitigated stigma included psychosocial support and survivor advocacy.

**Conclusions:**

Ebola‐related stigma complicates outbreak control and has adverse psychosocial effects that linger long after the outbreak is declared over. We provide a range of multilevel strategies for reducing stigma, including engagement with trusted community leaders, survivor‐centred support systems and provision of psychological support for responders.

## 1 Background

Ebola disease is a high‐fatality and often stigmatised disease. In September 2022, an Ebola outbreak caused by the Sudan ebolavirus was first identified at Mubende Regional Referral Hospital in central Uganda [1]. The outbreak resulted in 164 cases (142 confirmed, 22 probable), 87 recoveries and a case fatality ratio of 47% [1]. These numbers reflect the outbreak's clinical severity and the current absence of a definitive antiviral treatment for Sudan ebolavirus. Recovery is possible and can be improved with high‐quality supportive care [1, 2]; however, it is often accompanied by a substantial psychological and social toll [3, 4]. Many survivors endured not only the physical effects of the disease but also long‐term consequences of associated stigma.

Stigma is characterised by prejudiced attitudes and negative behaviours toward individuals or groups with traits considered discrediting or shameful in their society [5]. Ebola‐related stigma following the 2014–2016 West Africa epidemic resulted in ostracism, abuse and diminished quality of life of survivors [3, 6, 7, 8].

Stigma has also been repeatedly identified as a factor contributing to the undetected spread of ebolaviruses, as it encourages concealment of symptoms, delayed healthcare seeking and migration from high‐incidence communities [4, 6, 7, 8, 9]. The stigmatisation of outbreak response workers further hinders outbreak control efforts, as seen in the West Africa outbreak, where fear, disease denialism and stigma were thought to contribute to the death of eight healthcare workers during a health education campaign [10, 11, 12].

Although Ebola‐related stigma tends to decline over time, it can persist for years, affecting social integration, mental health and economic opportunities for survivors and their communities [8, 13, 14, 15, 16, 17, 18, 19]. For example, in the 2021 Ebola outbreak in Guinea, the link to semen from a survivor of the 2014 West Africa outbreak triggered a resurgence in stigmatisation of survivors [18, 20].

Despite evidence of the detrimental effects of Ebola‐related stigma in other contexts, research on Ebola stigma in the context of the 2022 Uganda outbreak remains very limited. A structured analysis of the relationship between Ebola stigma and outbreak control is also missing from the literature, despite the profound implications for future outbreaks.

This study aimed to explore how stigma emerged and manifested during the 2022 Sudan ebolavirus outbreak in Uganda and examine its impact on outbreak control efforts. The purpose of the study was to generate improved understanding of the processes involved in Ebola stigma, in order to inform the development of targeted stigma reduction interventions in future outbreaks.

## 2 Methods

### 2.1 Study Design, Setting and Participant Recruitment

For this qualitative study, we conducted in‐depth interviews with key informants involved in the Sudan ebolavirus outbreak in central Uganda.

We adopted maximum variation purposive sampling to ensure all locally identified key roles in outbreak response were represented. This included frontline healthcare workers, healthcare service managers, ministry of health representatives, psychosocial support team members, survivor programme staff, burial team members, village health team members and Ebola survivors. We identified potential participants through institutional contacts and snowball sampling and recruited participants via email invitation. All 12 key informants who were successfully contacted agreed to participate and were subsequently interviewed.

After ensuring inclusion of at least one representative from all identified key groups, the sample size was determined by data saturation—the point at which no new thematic sub‐domains emerged.

### 2.2 Data Collection

A researcher trained in qualitative methods, and without prior relationship with the participants (the first author), conducted once‐off, one‐on‐one interviews via Microsoft Teams using a topic guide. Participants were in private spaces either at their place of work or at home. The topic guide was informed by prior research and informal discussions with local stakeholders. Interviewees were briefed on the research objectives prior to giving informed consent. A verbal consent process was followed due to logistical considerations and the use of virtual interviews. Interview questions broadly focused on (1) what stigma manifestations were observed during or following the Ebola disease outbreak; (2) what contextual factors, beliefs or feelings contributed to this stigma; (3) how the stigma impacted on outbreak control and (4) suggestions for future stigma monitoring and reduction. The interviews, conducted between September 2023 and June 2024, lasted 25–50 min. With consent, we audio‐recorded and transcribed the interviews verbatim. Transcripts were not returned to the interviewees but were reviewed by the first author for consistency with the recordings.

### 2.3 Data Analysis

We uploaded the transcripts to NVivo Qualitative Data Analysis Software Release 1. Following familiarisation with the content, two researchers (the first and third authors) independently coded the transcripts using an iteratively adapted codebook. Transcripts were double coded until all inconsistencies were resolved. The remaining transcripts were then split equally between the coders. The research team met weekly while conducting interviews and analysing data to discuss emergent findings.

**FIGURE 1:**
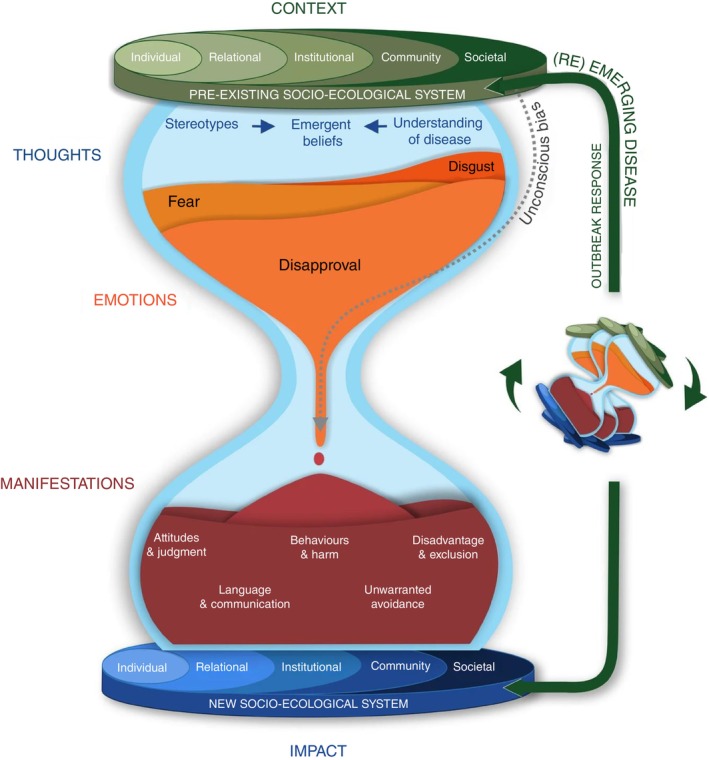
The hourglass stigma model. Reproduced from Paterson et al. [22] under CC BY licence.

We employed a combination of deductive and inductive framework analysis to ensure that the analysis was both theoretically grounded and open to emergent themes [21]. This approach is well‐suited for exploring a recognised health‐related social phenomenon like stigma in a novel context, as it balances prior theoretical understanding with the flexibility to capture new insights [21]. This case study was part of a larger project aimed at developing a conceptual understanding of stigma in the context of new and re‐emerging infectious disease outbreaks [22]. We adopted the hourglass stigma model, developed in the broader project, as the a priori conceptual framework for deductive framework analysis (Figure 1) [21, 22]. We inductively added sub‐themes that were not captured by the existing framework. Three participants reviewed the findings to confirm they accurately reflected the interview content.

### 2.4 Reporting Standards

We have reported our methods and findings according to the consolidated criteria for reporting qualitative research (COREQ) checklist (Supporting Information) [23].

## 3 Results

We conducted 12 interviews before reaching data saturation. The characteristics of the interviewees are detailed in Table 1.

**TABLE 1:** Interviewee characteristics.

Interviewee characteristics	*n* (%), *N* = 12
Roles in outbreak response^a^	
Frontline healthcare worker	6 (50)
Response coordinator/lead/trainer	3 (25)
Psychosocial support team member	3 (25)
Ebola survivor	2 (17)
Survivor programme staff	2 (17)
Peer support provider	2 (17)
Ministry of health representative	1 (8)
Healthcare service management	1 (8)
Village health team member	1 (8)
Burial team member	1 (8)
Involvement in previous Ebola disease outbreaks	
Yes	3 (25)
No	9 (75)

^a^ Interviewees often held multiple roles. To preserve clarity and anonymity, simplified unique identifiers are used in quote attributions and may not reflect the full scope of an individual's roles.

The key findings are detailed below, organised by stigma domain.

### 3.1 Domain I: Contextual Factors

Pre‐existing contextual factors considered drivers of Ebola stigma included political tensions and mistrust, witchcraft narratives and limited disease knowledge. Conversely, contextual factors such as prior outbreak experience and practices of collective caregiving were considered protective. These factors are further detailed in Table 2 with illustrative quotes.

**TABLE 2:** Pre‐existing contextual drivers and mediators of stigma.

Stigma drivers/facilitators	Protective/mediating factors
Societal	
*Coloniality* “By design the African population has been conditioned to trust fair‐skinned people. By design.”—Healthcare service manager *Mistrust of authorities* “Initially people believed they were being rounded up for other reasons. And you know, nothing to do with their health.”—Clinical lead and trainer	*Increased outbreak awareness* “One thing I noticed is the stigma was more with the earlier outbreaks than with subsequent outbreaks, somehow we're having less and less stigma. People now became more empathetic and they were understanding that people don't [always] get infected. Healthcare workers can work there and as long as they have maintained their infection prevention and control measures, they can still be safe.”—Clinical lead and trainer
Community	
*Limited available health information* “We as the doctors, our profession, we've not done a lot about community education, really.”—Ebola survivor and frontline healthcare worker *Witchcraft narratives* “Our communities have a lot of myths and everything, they believe these people, they were bewitched. Maybe they stole someone's goats.”—Ebola survivor and frontline healthcare worker	*Communal faith systems* “The people supported me spiritually. You know, you call me in the morning and pray for me and assure me that I will be fine.”—Ebola survivor, peer support lead *Collective care provision* “The African way of doing things is if you lose your parents then somebody like an uncle actually takes you in to live as his child.”—Clinical lead and trainer
Institutional	
*Resource limitations* “We can be two people and manage an [emergency room] that has over 20 beds and some patients are even on the floor. So a patient can come there, they don't get quality services … they say the doctor has failed, so they resort to the herbalist.”—Ebola survivor and frontline healthcare worker	*Trust in healthcare workers* “Someone from the community who doesn't have medical knowledge, he's trusting everything in the doctor, he knows that the doctor is doing his best to save them.”—Ebola survivor and frontline healthcare worker *Willing local clinical leadership* “We are soldiers in public health. And anything that happens, we are ready.”—Survivor programme lead
Relational	
*Shared narratives of harm* “Sometimes we will get situations where someone adversely reacts to a vaccine and that happens like 1 in 1,000,000, you know. But then that will become their point of attachment.”—Ebola survivor and frontline healthcare worker	*Phone contact* “They weren't allowed to visit. So I was there alone and I think the good thing is we had gone with the phone, so we were in touch.”—Ebola survivor, peer support lead
Individual	
*Limited disease knowledge* I think the underlying cause [of stigma] is individual lack of information and facts about Ebola.—Clinical and psychosocial support coordinator	*Stigma resilience through sense of duty* I was chosen to be a VHT, come rain, come shine, I had to go.—Village health team member

*Note*: Table organised according to socio‐ecological sub‐domains [24].

Abbreviations: ETU = Ebola treatment unit; VHT = village health team.

Interviewees explained that there were also characteristics specific to the disease and outbreak response interventions that both precipitated and prevented stigma (Table 3).

**TABLE 3:** Outbreak‐specific drivers and mediators of stigma.

Stigma drivers/facilitators	Protective/mediating factors
Pathogen‐related	
*Illness sequelae* “Some people needed readmission following discharge, especially the males who had painful scrotal swelling … And the community is looking at you, and so they doubt, did you bring us healed people? … Why are you taking them back?”—Ebola survivor and frontline healthcare worker	*Familiarity of pathogen* “We have responded many times. So there was a bit of comfort that we're not going to die, you know.”—Ministry of health representative
Response‐related	
*Delays in reporting* “We don't report so fast somehow. I think that's one of our biggest problems. So by the time it's recorded nationally or at the centre, there's been a lot of fear. There've been quite a number of deaths. There've been some myths that are associated with the illness and the deaths.”—Ministry of health representative	*Dedicated psychosocial support team* “The psychosocial team used to come three times in a day. They come in the morning, they pray for you. They assure you … and you become friends. They tell you to stop thinking this and that.”—Ebola survivor, peer support lead
*Visible burials* “Safe and dignified burial helped a lot to actually cut the chain of transmission. But it also came with a challenge where now if somebody came and did safe and dignified burial at a certain homestead then everybody in that homestead would be considered to be a sick person with Ebola.”—Clinical lead and trainer	*Accessible rumour‐dispelling* “I'm happy the Ugandan approach was good. We had the district task force … who had opportunity to regularly have talk shows on the radios. Of course, not everybody has a radio, but they were able to pass information on a consistent basis and dispel some rumours.”—Clinical and psychosocial support coordinator
*Lack of psychological support for responders* “Also the lack of a supportive care system for health care workers. There was no mental health care for the responders themselves. So that also needed to be found.”—Clinical and psychosocial support coordinator	*Purposeful physical contact between healthcare workers and survivors* “They [the healthcare workers] were with me. They even did the handshake when the other village mates were seeing. We even sat together on a bench with them. So this is when people, I think, started picking up that if these health workers can sit with him, they can have a handshake with him, then that's meaning he could be Ebola free.”—Ebola survivor, peer support lead
*Conspicuous survivor follow up* “The survivors feel the follow up is enough based on the fact that the neighbours still wonder, are these people still sick? Were they returned when they are sick? Or why are they still following them? … So that stigma still goes on.”—Psychosocial team member	*Peer support* “Now I told [the wife of a symptomatic community member], please let the husband go. Because me, myself, I went. I never thought that I would be back, but now that I'm back, this is an experience that you should also use … Everything is well in the ETU.”—Ebola survivor, peer support lead

Abbreviation: ETU = Ebola treatment unit.

### 3.2 Domain II: Thoughts and Beliefs

A range of misconceptions about Ebola disease arose as news of the outbreak spread. Interviewees noted that these beliefs were often rooted in mistrust of authority figures and institutions. Many recounted beliefs attributing the outbreak to government‐related conspiracies. This mistrust extended to Ebola response teams, with a wide range of narratives about the underlying motivation, including suggestions that people were being sent to treatment units to have their organs harvested or their land seized. These rumours appeared to fuel fear and reinforce stigma within communities.

You know, there are now a lot of myths and misconceptions about admissions or quarantine … like maybe they are going to extract organs.—Survivor programme lead

They feared that it was just propaganda, this whole epidemic … We saw many referring to this as an internal game, designed to impoverish them and seek their properties.—Healthcare service manager

Misunderstandings about recovery also contributed to stigma. Interviewees explained that public health messages explaining that there was no definitive antiviral treatment for Ebola disease were often misinterpreted to mean that recovery was impossible, discouraging individuals from seeking care. It was clear, however, that the relationship between knowledge of the disease and stigma was not linear. In some cases, specific pieces of knowledge, such as the persistence of the virus in semen, were misinterpreted or used in ways that contributed to targeted stigma:

The community learned that there can be persistence of the virus in semen. There was a threat passed over the local radio that if we have any new case of Ebola in the community, we will know it has come from the male survivors and we shall kill all of them.—Clinical and psychosocial support coordinator

Additionally, the link between Ebola disease and eating wild animals such as monkeys, often mentioned in education campaigns, was thought to deepen the social alienation of survivors in communities where these practices were considered taboo.

Because [Ebola is] associated with a lot of things like you're eating wild animals, you're eating monkeys, which we don't eat in this society … So that all comes with a lot of stigma. So when they look at you, you have Ebola, they straight away think maybe you ate a monkey.—Ebola survivor and frontline healthcare worker

### 3.3 Domain III: Emotional Responses

Fear emerged as the predominant emotional driver of Ebola stigma. All interviewees relayed stories of witnessing this fear in affected communities. A burial team member described arriving for burials in hazmat suits with community members fleeing at their approach. In some cases, outbreak response measures were thought to exacerbate this fear.

I think what makes the stigma worse is the way they come and pick you up in the community, like the ambulance, the health workers, they will come and spray the whole area. They want everyone … the whole community turns against you, you know, they're scared. When they search for your contacts, all these people are trapped, and they are looking for them … it alarms the community.—Ebola survivor, peer support lead

### 3.4 Domain IV: Stigma Manifestations

Reports of stigma ranged from negative attitudes to acts of physical harm (Table 4). Stigma was felt to extend beyond those who had the illness to anyone associated with them or the disease, including family members and healthcare workers. The enduring nature of Ebola stigma was an ongoing concern among interviewees:

The stigma spans from all the way from when they just suspect you have the disease. And then after when you go back to the community … Even if you get better, still, it's not the same.—Ebola survivor and frontline healthcare worker

**TABLE 4:** Illustrative examples of stigma by manifestation.

*Attitudes and judgement*
“But then after your recovery, when you're back as a doctor, they see you differently. Someone doesn't even feel very comfortable with you holding their child.”—Ebola survivor and frontline healthcare worker
*Language and communication*
“People in the community started calling us Ebola, like they'd call me [name] Ebola. So I got used to that. It's even become my name.”—Ebola survivor, peer support lead
Physical harm
“There was an incident that we went to some village and people were so brutal to us in that we had to come back and look for security first.”—Burial team member
*Unwarranted avoidance*
“On one of the visits, one of the family members took me aside and asked me, ‘do you think … are you okay to touch him?’ This was a survivor, who was their child … That rang in my ears.”—Survivor programme lead
*Disadvantage and exclusion*
“The kids that have come from a home that had an Ebola patient, they deny them school. An entire institution that should have knowledge says that, no, we don't want this child back at this school.”—Ebola survivor and frontline healthcare worker

### 3.5 Domain V: Stigma Impacts

#### 3.5.1 Individual Impact

Respondents detailed how Ebola stigma had lasting effects on survivors' mental well‐being and social connectedness. Stigma also had economic impacts, including the loss of livelihoods following recovery due to avoidance of businesses:

When I came out of the [Ebola treatment unit], I think it took me almost six months for customers to start turning up like before … For a week I was just opening, and I couldn't get anyone buying anything.*—*Ebola survivor, peer support lead

#### 3.5.2 Relational Impact

Stigma driven by fear of ongoing infectiousness was noted to fracture family structures and disrupt support networks. This rejection was particularly distressing for survivors who anticipated emotional support upon their release:

They told me my results were back, I'm going to be released from the [treatment unit]. So I took my phone, obviously I called my family members, my friends, even my fellow workmates … So when I came back home, I couldn't believe that I never found anyone home. Everyone at home had left.*—*Ebola survivor, peer support lead

#### 3.5.3 Institutional Impact

Stigma in healthcare settings meant that survivors often required ongoing dedicated services for routine medical concerns. Response coordinators warned that this additional strain on health resources was unsustainable.

Whenever [Ebola survivors] would go to the health centre to seek treatment, nobody would want to do anything with them. So they would have to come back to the isolation unit where we will treat them for simple things like malaria, respiratory tract infections.—Clinical and psychosocial support coordinator

More broadly, stigma was observed to exacerbate community mistrust of other public health interventions, including vaccination efforts:

Covid vaccination campaigns were tried during the Ebola outbreak. And the turn up was extremely low, because people were thinking that, ‘oh, there is Ebola now, and now you're bringing us a Covid vaccine, so you want to kill us’.*—*Ebola survivor and frontline healthcare worker

#### 3.5.4 Community Impact

At the community level, interviewees described persistent fears and avoidance behaviours in relation to survivors or others associated with the disease, particularly around physical proximity and shared spaces. This included avoidance in markets, places of work and social gatherings and often led to overt forms of ostracisation:

Some of these survivors who have come back home are still stigmatised, and they seem not to be very free in the community. At work, in the markets, or when they go into the shop to buy things, people keep pointing at them [saying] ‘see that one’.—Frontline healthcare worker and mental health lead

#### 3.5.5 Societal Impact (Outbreak Control)

By undermining preparedness, response and recovery efforts, stigma was believed to exacerbate the spread of Ebola disease, representing an important societal impact. Table 5 details how stigma affected all components of outbreak response. For example, community reluctance to engage in surveillance efforts, due to fear of discrimination, delayed case identification and treatment. Additionally, local healthcare workers faced stigma within their communities, leading to staff shortages that further strained the response.

**TABLE 5:** Impact of stigma on the WHO components of health emergency prevention, preparedness, response and resilience.

*Reluctance regarding collaborative surveillance*
“The communities used to turn on the surveillance teams as they go out, and the emergency medical teams as they go to pick up suspects in the communities … they would throw stones at the ambulances.”—Clinical and psychosocial support coordinator
*Compromised community protection due to impaired infection control measures*
“The stigma has a very big impact on the control of the outbreak in that if people are stigmatised, they will come late for diagnosis and isolation. And so they will be transmitting the infection because they have symptoms within their community, and that will prolong the control of the outbreak. Now, for us to control outbreak quickly, we need to do a lot on strategies to reduce stigma.”—Frontline healthcare worker and mental health lead
*Staffing shortages for safe and scalable care*
“The stigma can have impact on the people who are frontliners. The team who are responding. But in the community, if I'm not at the treatment unit. And I'm coming back to join my fellow colleagues. And I'm being stigmatised. Do you think next time I'll go back if I have not been empowered?”—Frontline healthcare worker and mental health lead “I have a healthcare worker who kept away from the hospital … a nutritionist and nutrition was one of the key components for case management … So yeah, stigma has an impact on epidemic response.”—Healthcare service manager
*Increased social barriers to accessing countermeasures*
“There are those before I left who were expressing the signs. And these were contacts already, I knew, I told them let's go to the hospital together. They said no, we cannot go there. How will people see it that we are in the hospital because of Ebola? … So these people, just because of stigma, they never wanted to turn up and show up that were Ebola contacts.”—Ebola survivor, peer support lead “There are families where they don't allow you to be vaccinated and not because they have gotten any adverse effect, but probably they feel like you're injecting them something that you want to kill them. Whoever makes the vaccine wants to kill them.”—Ebola survivor and frontline healthcare worker
*Emergency coordination challenges arising from workforce hesitancy*
“The moment it was confirmed an Ebola case, there was an explosion. Health workers literally took cover. They retreated … And they needed quick, quick, quick leadership to get them organised and [mobilise] them into responding effectively. So the stigma did come in immediately, but it was implied in the patients who came in who were not attended to immediately because of the fear.”—Healthcare service manager “In the first phase even health workers will not want to come near the ETU. But with time we tried to bring them on board. But even here, I was disappointed that the core health workers in [specific affected areas] did not actually take charge of the response … But I saw as the other experts from other places came and took over care from beginning to end, which for us in response [coordination], is not good. One day, people on the ground will take charge. The people who know the community.”—Clinical responder and coordinator

*Note*: Based on World Health Organisation. Strengthening the global architecture for health emergency prevention, preparedness, response and resilience [25].

Abbreviation: ETU = Ebola treatment unit.

### 3.6 Recommendations for Stigma Reduction

Interviewees offered a wide range of suggestions for reducing stigma in future Ebola disease outbreaks, many of which are transferable to other diseases. They particularly emphasised the importance of culturally appropriate interventions, community‐wide support and upholding the dignity of symptomatic individuals to minimise stigma and enhance response efforts (Table 6).

**TABLE 6:** Recommendations for stigma reduction improvement in future outbreaks.

Recommendation	Key quotes
1. Ensure risk communication is locally appropriate/led and ongoing	“I think crafting messaging that is appropriate, easily understandable in the locally spoken languages goes a long way in minimising stigma. And also having engagement of ongoing nature because it's not enough for you to do a community drive in a community and consider that you have covered that community and the stigma levels have been reduced.”—Clinical and psychosocial support coordinator
2. Engage local leaders and institutions early	“They need to reach out to schools. I must say that. During the outbreak, there was one student … he was negative two times, and when we took him back to the school, the whole school ran away from him. And that wasn't good. Including the teachers. So institutions of learning and others must not be left aside.”—Frontline healthcare worker and mental health lead “Engaging other leaders, especially the leaders that the communities believe in, opinion leaders, like local council leaders, like religious leaders, like witch doctors or traditional healers. These go a long way in assuring the community and raising their comfort levels and thereby also reducing stigma.”—Clinical and psychosocial support coordinator
3. Include anti‐stigma messaging in community health education	“When you're doing the community health education, try to also bring in the psychosocial bit of it so that people can be prepared mentally to get sick or receive [a recovered person].”—Ebola survivor and frontline healthcare worker
4. Minimise attention during survivor follow ups	“Usually in Ebola, the organisations bring very good new cars, Land Cruisers. And so, when Land Cruisers go to visit a particular person in the community, it arouses a lot of concern, and we should minimise it as much as possible … If we have to go there, go there as nobody. You can park the vehicle somewhere and walk to that place and, also, wear normal clothes. We had our uniforms and jackets and all those things they associate with Ebola. I think it is important to decrease stigma that we should make our workers as normal as possible.”—Clinical responder and coordinator
5. Extend recovery efforts to the full community	“We should also try to involve the other people who are not infected. Everybody, for example, in Mubende, even those who were not infected, in a way they were affected. And so when addressing this issue, we don't need to leave them behind … bring programs that will support all the people who are affected.”—Frontline healthcare worker and mental health lead
6. Offer psychological support for responders	“The psychosocial team sometimes they are overwhelmed and we underestimate their contribution to the outbreaks.”—Frontline healthcare worker and mental health lead “I think maybe the ministry should take [supporting outbreak responders] more seriously. And it should be having some support to give to those people because there's much torture* in the community when you take the project.”—Burial team member *The speaker's use of the term “torture” here is understood to refer to the social, emotional, and psychological burden associated with outbreak response work.
7. Optimise privacy provisions when doing case detection	“Maybe [the whole community] should not know about what is taking place. Not until maybe I'm a confirmed case because I can come back when I'm not confirmed. A negative case. And then the information has spread all over that I'm confirmed. I would prefer… they can ask me, in which comfortable place can we find you and pick you up? I tell you … Then when I'm taken, they can come and ask my relatives or ask me, myself, or try to intervene. But if I'm confirmed now and I'm sitting here and then, in front of the community, they must come and stand here, and take me when everyone is seeing. Even I got scared of it.”—Ebola survivor, peer support lead
8. Conduct detailed stigma and rumour tracking	“I think we need to have some metrics, a stigma index … to be able to tell at the community level, for instance, objectively what's the level of stigma and be able to measure it over time.”—Clinical and psychosocial support coordinator “I think it is important to continue to track rumours because you go dispel one myth and before you know it, another one is coming up.”—Clinical and psychosocial support coordinator
9. Prioritise holistic and palliative care of patients	“We need to try our level best to ensure that the patients who come to us are at an early stage … But also in the care of the patients, we must not only focus on the Ebola virus, there could be other action [especially when] so many of the patients are dying.”—Frontline healthcare worker and mental health lead
10. Facilitate survivor peer support and advocacy	“The other point that we need to focus on is using the survivors a lot more [and let them share] about their experience. And that in a way, will help us to reduce all stigma.”—Frontline healthcare worker and mental health lead
11. Facilitate early involvement of psychosocial team	“The psychosocial base right from the beginning of testing someone, that is one thing we should do to reduce the stigma. The people need to be talked to, and before they are tested.”—Frontline healthcare worker and mental health lead
12. Ensure responders are paid in a timely manner and adequately protected	“There was also a fear that people would not be facilitated with risk allowances, they would not be paid. And this actually came to pass where a good number of people were not paid. So in terms of participation in the response, I think it was … about the ability to be supported with adequate resources for protection.”—Clinical and psychosocial support coordinator

## 4 Discussion

### 4.1 The Emergence of Ebola Stigma

The findings of this study highlight how stigma during the Sudan ebolavirus outbreak in central Uganda was driven by a complex interplay of pre‐existing and emergent contextual factors. Emergent factors included public health response efforts, which inadvertently fostered stigma. This finding is consistent with observations from the country's 2000/2001 Ebola outbreak, where outbreak control strategies that induced excessive fear were identified as a key driver of stigma [26]. The handling of previous infectious outbreaks in the region may have also contributed to the mistrust in leadership and treatment units, which emerged strongly in our findings [26].

A lack of knowledge about Ebola disease transmission and management also contributed to stigma. Our findings suggested that this gap in information often allowed misconceptions of the illness to form and spread. The role of disease misconceptions in fostering prejudice and discrimination is well‐recognised [27]. However, interviewees noted that community education, if not paired with anti‐stigma messaging, did not inherently decrease stigma. For example, raising awareness about the risk of ongoing semen transmission without addressing stigma unintentionally heightened fear, as was the case in Guinea [18, 20]. These findings are consistent with stigma patterns observed in relation to other infectious diseases, such as HIV, COVID‐19 and mpox, where risk communication has sometimes inadvertently reinforced stigma when not accompanied by efforts to address fear, blame and moral judgement [28, 29, 30, 31].

Personal resilience has been reported as a protective factor against stigmatisation in other Ebola‐affected countries [32, 33]. In our study, this was particularly evident among healthcare workers, who found meaning in their contributions to the outbreak response and empathised with community fears. Psychosocial support, including peer networks and communal faith systems, played a crucial role in fostering coping strategies and promoting recovery.

### 4.2 The Lasting Impact of Ebola Stigma

Ebola stigma has been shown to significantly impact affected individuals' mental health, contributing to depression, anxiety, post‐traumatic stress disorder and suicide [16, 18, 33, 34, 35, 36]. In addition to exacerbating psychological trauma, stigma also heightens social vulnerability, due to the socio‐economic ramifications [37]. Our findings corroborate this, with survivors reporting family rejection and economic hardship due to perceptions of lingering contagion. Participation in outbreak response efforts, such as peer support or community education, has previously been shown to aid survivors in rebuilding livelihoods [38]. This is supported by our data. Social protection programmes, like those implemented in Sierra Leone, have similarly demonstrated positive effects on wellbeing [39], underscoring the importance of accessible psychosocial support during and after treatment to mitigate long‐term stigma impacts.

### 4.3 Stigma and Outbreak Control

Our findings illustrate how, once stigma had taken hold, it undermined outbreak control measures. Reluctance to participate in contact tracing, concealment of symptoms and poor adherence to burial practices were widely reported in our study, consistent with prior evidence [40, 41, 42]. Commonly cited concerns about outbreak control efforts, including loss of privacy, civil liberties and culturally important closure, were also echoed by interviewees [5, 40]. Healthcare worker morale was negatively affected, with reports of insufficient support, training and protections making it more difficult to endure the stigma associated with outbreak response efforts [43]. Taken together, our findings reinforce the bidirectional relationship between stigma and outbreak control, highlighting its role in prolonging transmission and increasing mortality risks.

### 4.4 Recommendations for Stigma Reduction

Several interventions implemented during the outbreak were considered effective in reducing stigma and should be prioritised in future responses. These include the early involvement of psychosocial support teams, establishing survivor support groups from the onset of the outbreak and facilitating safe contact between inpatients and families, such as through mobile phones. Outbreak control measures should prioritise preserving patients' dignity and self‐determination without compromising public health. This includes ensuring privacy in contact tracing and safe and dignified burials to encourage participation without fear of judgement [44, 45]. Improving the design of treatment facilities or offering a community‐based isolation strategy for contacts, as successfully piloted during the 2018–2020 DRC outbreak, could also help address stigma [44]. Finally, strengthening care through adequate training, timely payments, proper protection and psychological support for responders is essential to sustain morale, ultimately fostering community trust and reducing stigma [46].

### 4.5 Strengths and Limitations

To our knowledge, our study is the first structured investigation into the facilitators and outbreak control impacts of Ebola stigma in Uganda. A wide range of key stakeholders were included in this study, allowing for varied perspectives to be captured. The use of a conceptual model that accounts for socio‐ecological systems, rather than just individuals' knowledge, attitudes and behaviours, enabled more nuanced and actionable findings to be reported. The research team was independent from local government institutions, thus reducing the risk of institutional mistrust impacting the findings.

Limitations of the study include the use of Microsoft Teams for conducting interviews, which may have excluded stakeholders without internet connection. Participants were restricted to those involved in the outbreak response and therefore only indirectly capture views from the lay community. All interviewees were also aware of the topic of discussion and, therefore, may have been inclined to emphasise observations and stigma experiences. The time elapsed between the Ebola outbreak and interviews may have resulted in recall bias.

### 4.6 Future Research

Longitudinal studies are required to monitor Ebola stigma in affected communities and to evaluate the effectiveness of stigma reduction interventions in future outbreaks.

## 5 Conclusion

Stigma during the Ebola disease outbreak in central Uganda was shaped by a combination of pre‐existing social dynamics and new challenges introduced by outbreak response measures. The resulting cascade of negative beliefs, emotions and behaviours created lasting impacts across individuals and communities, undermining both mental health and outbreak control efforts. A central finding of this study is that response measures that create a sense of ‘otherness’ can unintentionally deepen stigma, complicating outbreak control. By amplifying the voices of local responders and survivors, this research offers practical insights and recommendations for reducing stigma in future outbreaks, with lessons that extend beyond Ebola disease.

## Ethics Statement

This study was approved by the University of Oxford's Medical Sciences Division Research Ethics Committee (reference R87073/RE001).

## Conflicts of Interest

The authors declare no conflicts of interest.

## Supporting information


**Data S1:** COREQ Checklist.

## Data Availability

The interviews from this study are not available due to the risk of re‐identification.
